# Synthesis and Activity of Novel Acylthiourea with Hydantoin

**DOI:** 10.3390/ijms141019526

**Published:** 2013-09-26

**Authors:** Jintao Han, Hongbo Dong, Zhihong Xu, Jinmin Wang, Mingan Wang

**Affiliations:** 1 Department of Applied Chemistry, China Agricultural University, Beijing 100193, China; E-Mails: hanjint321@163.com (J.H.); bloodwhenseeme@163.com (H.D.); x_u_78@sina.com (Z.X.); wangjinmin@126.com (J.W.); 2 College of Agricuture, Yangtze University, Jingzhou 434025, Hubei, China

**Keywords:** acylthiourea, hydantoin, thiohydantoin, herbicidal activity, fungicidal activity

## Abstract

The 41 novel acylthiourea derivatives with hydantoin were synthesized in moderate to excellent yields by using 5-(4-aminophenyl)- and 5-(4-aminobenzyl)- hydantoin or 5-(4-aminobenzyl)-thiohydantoin as raw materials and characterized by IR, ^1^H NMR spectra and elementary analysis. The preliminary bioassay showed that these compounds exhibit certain selectively herbicidal activities with the 91%, 94% and 87% inhibition rates of **7l**, **8o** and **8p** against *B. campestris*, 100%, 100% and 95% efficacy against *B. campestris* in a greenhouse test, respectively. **7a**, **7b**, **7c** and **7d** exhibited 74%, 79%, 79% and 71% inhibition rates against *F. oxysporum*, respectively.

## 1. Introduction

**Scheme 1:**
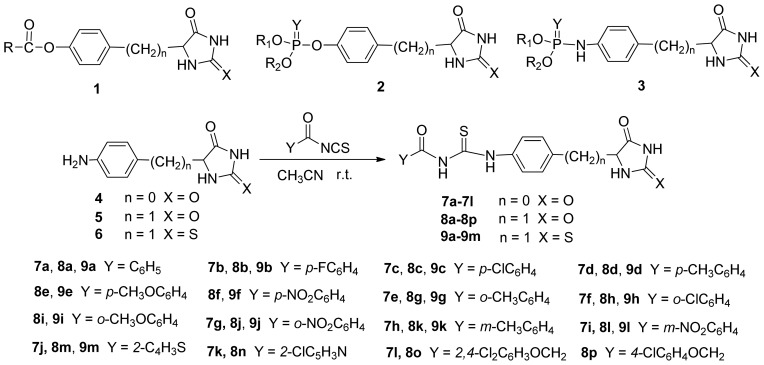
The synthetic route of new acylthiourea derivatives.

Hydantoin and thiohydantion are important core moiety in the design and synthesis of active molecules as well as natural products; these derivatives have not only been used in medicinal chemistry as anti-HSV, antidiabetic, HDL-cholesterol modulators, but also used as fungicides and herbicides in agrochemical research [1–6]. In addition, these derivatives are very useful building blocks for the synthesis of various heterocycles such as 5-arylidene derivatives of imidazoline-4-one, imidazothiazine, diazinone, and diazepinone [7–9]. In our laboratory, several types of hydantoin and thiohydantioin derivatives (**1**, **2**, **3**) were synthesized, and we showed that some of them exhibit good herbicidal, fungicidal and insecticidal activities [10–13]. Acylthioureas are the key structural motifs of numerous compounds that displayed a wide range of biological activity such as antimicrobial, antipathogenic, insecticidal, fungicidal, antitumor activities and influenza virus inhibitors [14–21]. Acylthioureas are widely used as the building blocks for the synthesis of various heterocycles such as thiazolidene-2-imine, imidazole-2-thione and 2-thioxo-4-imidazolidinone [22–33]. We could not find acylthiourea derivatives containing hydantoin and thiohydantion heterocycles in references. Continuing effort of our ongoing project aimed at looking for novel biologically active hydantoin and thiohydantion heterocyclic compounds [10–13], herein, we report a new type of acylthiourea derivatives containing hydantoin and thiohydantion heterocycles (Scheme 1) and their biological activities.

## 2. Results and Discussion

In our laboratory, we synthesized 5-aryl derivatives of hydantoin and thiohydantoin to develop the novel inhibitor of Adenylosuccinate Synthetase (AdSS) [10–13], which plays a key role in the two-step conversion of IMP to AMP in the *de novo* pathway of purine biosynthesis [34–36]. Some of the esters **1** were tested and showed strong herbicidal activities against *Zea mays*, *Triticum aestivum* and *Arabidopsis thaliana*, the further greenhouse test showed that compounds have 60%, 50% and 50% efficacy against *Stellaria media*, *Echinochloa crus-galli and Setaria viridis* at the dosage of 1000 g/ha when used as a pre-emergence treatment, respectively [13]. After that, the thiophosphates **2** and phosphoramidates were found to show weak herbicidal activities against *Brasica campestris* and *Echinochloa crus-galli*, while one of them exhibited excellent insecticidal activities against *Myzus Persicae* [11,12]. Then, phosphoramides **3** were further tested and showed increasing herbicidal activities against *Brasica campestris* as well as insecticidal activities against *Myzus Persicae* [37]. These results indicated that 5-(4-aminophenyl)-hydantoin, 5-(4-aminobenzyl)-hydantoin and 5-(4-aminobenzyl)-thiohydantoin were important moiety for these active compounds. On the other hand, acylthiourea derivatives have been widely used and synthesized in medicinal chemistry and agrochemical research [14–21] in recent years. Based on these characters, we combined the containing-hydantoin amino derivatives and acylthioureas into a molecule and designed the novel acylthiourea derivatives containing hydantoin and thiohydantoin heterocycles (**7**, **8**, **9**). The synthesis was carried out by the reaction of 5-(4-aminophenyl)-hydantoin, 5-(4-aminobenzyl)-hydantoin and 5-(4-aminobenzyl)-thiohydantoin with the freshly prepared acyl isothiocyanates in moderate to excellent yields.

The data in Table 1 showed that some of these compounds, such as **7l**, **8o** and **8p** exhibit 91%, 94% and 87% inhibition rates against *B. campestris*, respectively, while they only have less than 25% inhibition rates against *E. crus-galli* at the concentration of 100 μg/mL. Further tests in a greenhouse were performed and the results in Table 2 showed that **7l**, **8o** and **8p** inhibit the growth of *B. campestris* with 100%, 100% and 95% efficacy after the post-emergence treatments, and only 23%, 46% and 31% efficacy after the pre-emergence treatments at the dosage of 1000 g/ha. However they exhibited less than 15% efficacy against *E. crus-galli* after the post-emergence or pre-emergence treatments. These results indicated that these compounds exhibit certain selectively herbicidal activities.

The data in Table 3 showed that only **7a**, **7b**, **7c**, **7d** and **7h** exhibit more than 70% inhibition rates against *Fusarium oxysporum*, and the others including **8** and **9** (data not shown in Table 3) have less than 55% inhibition rates against *Alternaria solani*, *Botryospuaeria berengeriana*, *Cercospora arachidcola* and *Fusahum graminearum* at the concentration of 100 μg/mL. The inhibition rates of **7a**, **7b**, **7c**, **7d** and **7h** against *F. oxysporum* were 74%, 79%, 79%, 71% and 71%, respectively. They are weaker when compared to carbendazin (the positive control) against *F. oxysporum* and need further structure modification to increase the fungicidal activity.

## 3. Experimental Section

### 3.1. General Information

All reactions were performed under room temperature with magnetic stirring. Unless otherwise stated, all reagents were purchased from commercial suppliers and used without further purification. Organic solutions were concentrated under reduced pressure using a rotary evaporator or oil pump. Melting points were measured on a Yanagimoto apparatus (Yanagimoto MFG Co., Kyoto, Japan) and uncorrected. Infrared spectra were recorded using a Shimadzu IR-435 instrument with KBr plates. ^1^H NMR spectra were obtained on Bruker DPX 300 spectrometer (Bruker Biospin Co., Stuttgart, Germany) with DMSO-*d*_6_ as a solvent and TMS as an internal standard. Elemental analysis was performed on a Vario EL instrument (Elementar Vario Micro Cube, Hanau, Germany).

### 3.2. Synthesis

#### 3.2.1. Synthesis of 5-(4-Aminobenzyl)-, 5-(4-Aminophenyl)-Hydantoin (**4** and **5**), 5-(4-Aminobenzyl)-Thiohydantoin (**6**) and Acyl Isothiocyanate Derivatives

The synthesis of the intermediates **4**, **5** and **6** were carried out according to the protocols in our previous paper and their spectral data were identical with that in the reference [37]. To a suspension of benzoic acid derivative (20 mmol) in 25 mL of CH_2_Cl_2_ in a 50 mL three-necked flask, 8 mL SOCl_2_ and a drop of *N*,*N*-dimethylformamide (DMF) were added. After stirring at room temperature for 3 h, the solution was evaporated. The resulting acyl chloride was dissolved in 15 mL of anhydrous acetonitrile and added to a solution of 20 mmol potassium thiocyanate in 25 mL of acetonitrile with two drops of polyethylene glycol-400 (PEG-400). After stirring at room temperature for 2 h, the mixture was filtered to give the acyl isothiocyanate derivatives, which were used without further purification [14,15].

#### 3.2.2. General Procedure for the Synthesis of Compounds **7**, **8** and **9**

To a stirred solution of 20 mmol 5-(4-aminophenyl)-hydantoin (**4**), or 5-(4-aminobenzyl)-hydantoin (**5**) or 5-(4-aminobenzyl)-2-thiohydantoin (**6**) in 20 mL of anhydrous acetonitrile, the acyl isothiocyanate solution in acetonitrile freshly prepared were added dropwise at ambient temperature. The reaction was monitored by TLC. After leaving it overnight, the reaction was stopped and the product was filtered. The products were further purified by recrystallization using DMF-EtOH-H_2_O to afford the compounds **7**, **8** and **9**.

*N*-((4-(2,4-dioxoimidazolidin-5-yl)phenyl)carbamothioyl)benzamide **7a**, white solid, yield 82%, m.p. 140–142 ºC, ^1^H NMR (DMSO-d_6_, 300 MHz) δ: 12.60 (s, 1H, NH), 11.58 (s, 1H, NH), 10.82 (s, 1H, NH), 8.43 (s, 1H, NH), 7.97 (d, *J* = 8.4 Hz, 2H, ArH), 7.73–7.52 (m, 5H, ArH), 7.38 (d, *J* = 8.4 Hz, 2H, ArH), 5.20 (s, 1H, CH); IR (KBr) ν: 3160, 3053, 1787, 1725, 1672, 1597 cm^−1^. Anal calcd. for C_17_H_14_N_4_O_3_S: C 57.62, H 3.98, N 15.81; Found: C 57.59, H 3.90, N 15.78.

*N*-((4-(2,4-dioxoimidazolidin-5-yl)phenyl)carbamothioyl)-4-fluorobenzamide **7b**, white solid, yield 70%, m.p. 146–148 ºC, ^1^H NMR (DMSO-d_6_, 300 MHz) δ: 12.55 (s, 1H, NH), 11.64 (s, 1H, NH), 10.83 (s, 1H, NH), 8.44 (s, 1H, NH), 8.09–8.03 (m, 2H, ArH), 7.70 (d, 2H, *J* = 8.4 Hz, ArH), 7.42–7.32 (m, 4H, ArH), 5.20 (s, 1H, CH); IR (KBr) ν: 3125, 3041, 1784, 1729, 1666, 1601 cm^−1^. Anal calcd. for C_17_H_13_FN_4_O_3_S: C 54.83, H 3.52, N 15.05; Found: C 54.80, H 3.50, N 15.01.

*N*-((4-(2,4-dioxoimidazolidin-5-yl)phenyl)carbamothioyl)-4-chlorobenzamide **7c**, white solid, yield 55%, m.p.230–232 ºC, ^1^H NMR (DMSO-d_6_, 300 MHz) δ: 12.50 (s, 1H, NH), 11.69 (s, 1H, NH), 10.81 (s, 1H, NH), 8.43 (s, 1H, NH), 7.99 (d, *J* = 7.4 Hz, 2H, ArH), 7.70 (d, *J* = 8.4 Hz, 2H, ArH), 7.62 (d, *J* = 7.4 Hz, 2H, ArH), 7.37 (d, *J* = 8.4 Hz, 2H, ArH), 5.20 (s, 1H, CH); IR (KBr) ν: 3145, 3035, 1783, 1728, 1670, 1593 cm^−1^; Anal calcd. for C_17_H_13_ClN_4_O_3_S: C 52.51, H 3.37, N 14.41; Found: C 52.53, H 3.41, N 14.36.

*N*-((4-(2,4-dioxoimidazolidin-5-yl)phenyl)carbamothioyl)-4-methylbenzamide **7d**, white solid, yield 53%, m.p.220–222 ºC, ^1^H NMR (DMSO-d_6_, 300 MHz) δ: 12.65 (s, 1H, NH), 11.50 (s, 1H, NH), 10.82 (s, 1H, NH), 8.44 (s, 1H, NH), 7.91 (d, *J* = 7.5 Hz, 2H, ArH), 7.71 (d, *J* = 8.4 Hz, 2H, ArH), 7.39–7.34 (m, 4H, ArH), 5.20 (s, 1H, CH), 2.40 (s, 3H, CH_3_); IR (KBr) ν: 3150, 3035, 1786, 1726, 1664, 1598 cm^−1^; Anal calcd. for C_18_H_16_N_4_O_3_S: C 58.68, H 4.38, N 15.21; Found: C 58.63, H 4.40, N 15.11.

*N*-((4-(2,4-dioxoimidazolidin-5-yl)phenyl)carbamothioyl)-2-methylbenzamide **7e**, white solid, yield 54%, m.p.138–140 ºC, ^1^H NMR (DMSO-d_6_, 300 MHz) δ: 12.62 (s, 1H, NH), 11.51 (s, 1H, NH), 10.82 (s, 1H, NH), 8.43 (s, 1H, NH), 7.84–7.70 (m, 4H, ArH), 7.47–7.37 (m, 4H, ArH), 5.20 (s, 1H, CH), 2.40 (s, 3H, CH_3_); IR (KBr) ν: 3170, 3045, 1789, 1715, 1677, 1598 cm^−1^; Anal calcd. for C_18_H_16_N_4_O_3_S: C 58.68, H 4.38, N 15.21; Found: C 58.62, H 4.55, N 15.35.

*N*-((4-(2,4-dioxoimidazolidin-5-yl)phenyl)carbamothioyl)-2-chlorobenzamide **7f**, white solid, yield 55%, m.p.222–224 ºC, ^1^H NMR (DMSO-d_6_, 300 MHz) δ: 12.34 (s, 1H, NH), 12.03 (s, 1H, NH), 10.83 (s, 1H, NH), 8.44 (s, 1H, NH), 7.72 (d, *J* = 7.4 Hz, 2H, ArH), 7.65–7.43 (m, 4H, ArH), 7.38 (d, *J* = 7.4 Hz, 2H, ArH), 5.21 (s, 1H, CH); IR (KBr) ν: 3301, 3056, 1783, 1715, 1694, 1593 cm^−1^; Anal calcd. for C_17_H_13_ClN_4_O_3_S: C 52.51, H 3.37, N 14.41; Found: C 52.60, H 3.41, N 14.44.

*N*-((4-(2,4-dioxoimidazolidin-5-yl)phenyl)carbamothioyl)-2-nitrobenzamide **7g**, white solid, yield 63%, m.p.234–236 ºC, ^1^H NMR (DMSO-d_6_, 300 MHz) δ: 12.25 (s, 1H, NH), 12.17 (s, 1H, NH), 10.84 (s, 1H, NH), 8.46 (s, 1H, NH), 8.23 (d, *J* = 8.1 Hz, 1H, ArH), 7.94–7.71 (m, 5H, ArH), 7.39 (d, *J* = 7.4 Hz, 2H, ArH), 5.22 (s, 1H, CH); IR (KBr) ν: 3146, 3034, 1764, 1716, 1690, 1593 cm_−1_; Anal calcd. for C_17_H_13_N_5_O_5_S: C 51.12, H 3.28, N 17.54; Found: C 51.14, H 3.21, N 17.25.

*N*-((4-(2,4-dioxoimidazolidin-5-yl)phenyl)carbamothioyl)-3-methylbenzamide **7h**, white solid, yield 54%, m.p.158–160 ºC, ^1^H NMR (DMSO-d_6_, 300 MHz) δ: 12.62 (s, 1H, NH), 11.54 (s, 1H, NH), 10.83 (s, 1H, NH), 8.45 (s, 1H, NH), 7.83–7.70 (m, 4H, ArH), 7.49–7.36 (m, 4H, ArH), 5.21 (s, 1H, CH), 2.39 (s, 3H, CH_3_); IR (KBr) ν: 3210, 3047, 1769, 1726, 1668, 1600 cm^−1^; Anal calcd. for C_18_H_16_N_4_O_3_S: C 58.68, H 4.38, N 15.21; Found: C 58.64, H 4.40, N 15.16.

*N*-((4-(2,4-dioxoimidazolidin-5-yl)phenyl)carbamothioyl)-3-nitrobenzamide **7i**, white solid, yield 52%, m.p.162–164 ºC, ^1^H NMR (DMSO-d_6_, 300 MHz) δ: 12.42 (s, 1H, NH), 12.04 (s, 1H, NH), 10.83 (s, 1H, NH), 8.78 (d, *J* = 2.1 Hz, 1H, ArH), 8.51–8.47 (m, 1H, ArH), 8.45 (s, 1H, NH), 8.37 (dd, *J* = 7.8, 2.4Hz, 1H, ArH), 7.84 (t, *J* = 7.8 Hz, 1H, ArH), 7.72 (d, *J* = 8.4 Hz, 2H, ArH), 7.39 (d, *J* = 8.4 Hz, 2H, ArH), 5.21 (s, 1H, CH); IR (KBr) ν: 3229, 3047, 1776, 1723, 1677, 1600 cm^−1^; Anal calcd. for C_17_H_13_N_5_O_5_S: C 51.12, H 3.28, N 17.54; Found: C 51.02, H 3.30, N 17.54.

*N*-((4-(2,4-dioxoimidazolidin-5-yl)phenyl)carbamothioyl)thiophene-2-carboxamide **7j**, white solid, yield 80%, m.p. 226–228 ºC, ^1^H NMR (DMSO-d_6_, 300 MHz) δ: 12.44 (s, 1H, NH), 11.64 (s, 1H, NH), 10.81 (s, 1H, NH), 8.43 (s, 1H, NH), 8.38 (d, *J* = 3.8 Hz, 1H, ThH), 8.05 (d, *J* = 5.0 Hz, 1H, ThH), 7.70 (d, *J* = 7.4 Hz, 2H, ArH), 7.36 (d, *J* = 7.4 Hz, 2H, ArH), 7.26 (dd, *J* = 3.8, 5.0 Hz, 1H, ThH), 5.20 (s, 1H, CH); IR (KBr) ν: 3138, 3043, 1778, 1722, 1657, 1592 cm^−1^. Anal calcd. for C_15_H_12_N_4_O_3_S_2_: C 49.99, H 3.36, N 15.55; Found: C 49.97, H 3.39, N 15.56.

2-Chloro-*N*-((4-(2,4-dioxoimidazolidin-5-yl)phenyl)carbamothioyl)isonicotinamide **7k**, white solid, yield 73%, m.p. 212–214 ºC, ^1^H NMR (DMSO-d_6_, 300 MHz) δ: 12.25 (s, 1H, NH), 11.96 (s, 1H, NH), 10.81 (s, 1H, NH), 8.62 (d, *J* = 5.4 Hz, 1H, PyH), 8.43 (s, 1H, NH), 7.99 (s, 1H, PyH), 7.84 (dd, *J* = 1.2, 5.4 Hz, 1H, PyH), 7.70 (d, *J* = 7.4 Hz, 2H, ArH), 7.39 (d, *J* = 7.4 Hz, 2H, ArH), 5.20 (s, 1H, CH); IR (KBr) ν: 3150, 3042, 1765, 1719, 1666, 1593 cm^−1^. Anal calcd. for C_16_H_12_ClN_5_O_3_S: C 49.30, H 3.10, N 17.97; Found: C 49.26, H 3.08, N 17.90.

2-(2,4-dichlorophenoxy)-*N*-((4-(2,4-dioxoimidazolidin-5-yl)phenyl)carbamothioyl)acetamide **7l**, white solid, yield 59%, m.p. 258–260 ºC, ^1^H NMR (DMSO-d_6_, 300 MHz) δ: 11.85 (s, 1H, NH), 10.77 (s, 1H, NH), 10.24 (s, 1H, NH), 8.37 (s, 1H, NH), 7.63–7.60 (m, 3H, ArH), 7.37 (dd, *J* = 2.4, 8.4 Hz, 1H, ArH), 7.27 (d, *J* = 7.5 Hz, 2H, ArH), 7.09 (d, *J* = 8.4 Hz, 1H, ArH), 5.11 (s, 1H, CH), 4.86 (s, 2H, CH_2_); IR (KBr) ν: 3288, 3060, 1780, 1740, 1682, 1600 cm^−1^. Anal calcd. for C_18_H_14_Cl_2_N_4_O_4_S: C 47.69, H 3.11, N 12.36; Found: C 47.58, H 3.12, N 12.34.

*N*-((4-((2,4-dioxoimidazolidin-5-yl)methyl)phenyl)carbamothioyl)benzamide **8a**, white solid, yield 93%, m.p. 240–242 ºC, ^1^H NMR (DMSO-d_6_, 300 MHz) δ: 12.61 (s, 1H, NH), 11.53 (s, 1H, NH), 10.48 (s, 1H, NH), 7.99–7.96 (m, 3H, ArH+NH), 7.70–7.64 (m, 3H, ArH), 7.57–7.52 (m, 2H, ArH),7.23 (d, *J* = 7.8 Hz, 2H, ArH), 4.37–4.34 (m, 1H, CH), 2.99–2.89 (m, 2H, CH_2_); IR (KBr) ν: 3172, 3064, 1766, 1726, 1668, 1597 cm^−1^. Anal calcd. for C_18_H_16_N_4_O_3_S: C 58.68, H 4.38, N 15.21; Found: C 58.98, H 4.30, N 15.25.

*N*-((4-((2,4-dioxoimidazolidin-5-yl)methyl)phenyl)carbamothioyl)-4-flurobenzamide **8b**, white solid, yield 83%, m.p. 216–218 ºC, ^1^H NMR (DMSO-d_6_, 300 MHz) δ: 12.56 (s, 1H, NH), 11.58 (s, 1H, NH), 10.48(s, 1H, NH), 8.09–8.03(m, 2H, ArH), 7.96 (s, 1H, NH), 7.64 (d, *J* = 8.0 Hz, 2H, ArH), 7.40–7.35 (m, 2H, ArH), 7.23 (d, *J* = 8.0 Hz, 2H, ArH), 4.37–4.34 (m, 1H, CH,), 2.97–2.94 (m, 2H, CH_2_); IR (KBr) ν: 3178, 3060, 1762, 1727, 1669, 1600 cm^−1^. Anal calcd for C_18_H_15_FN_4_O_3_S: C 55.95, H 3.91, N 14.50; Found: C 55.86, H 4.01, N 14.51.

*N*-((4-((2,4-dioxoimidazolidin-5-yl)methyl)phenyl)carbamothioyl)-4-chlorobenzamide **8c**, white solid, yield 63%, m.p. 232–234 ºC, ^1^H NMR (DMSO-d_6_, 300 MHz) δ: 12.51 (s, 1H, NH), 11.64 (s, 1H, NH), 10.49 (s, 1H, NH), 7.98–7.93 (m, 3H, ArH + NH), 7.66–7.56 (m, 4H, ArH), 7.23 (d, 2H, *J* = 8.4 Hz, ArH), 4.37–4.33 (m, 1H, CH), 2.95–2.93 (m, 2H, CH_2_); IR (KBr) ν: 3155, 3034, 1758, 1726, 1666, 1594 cm^−1^. Anal calcd. for C_18_H_15_ClN_4_O_3_S: C 53.67, H 3.75, N 13.91; Found: C 53.78, H 3.79, N 13.90.

*N*-((4-((2,4-dioxoimidazolidin-5-yl)methyl)phenyl)carbamothioyl)-4-methylbenzamide **8d**, white solid, yield 66%, m.p. 228–230 ºC, ^1^H NMR (DMSO-d_6_, 300 MHz) δ: 12.67 (s, 1H, NH), 11.43 (s, 1H, NH), 10.48 (s, 1H, NH), 7.96 (s, 1H, NH), 7.91 (d, *J* = 8.1 Hz, 2H, ArH), 7.65 (d, *J* = 8.4 Hz, 2H, ArH), 7.35 (d, *J* = 8.1 Hz, 2H, ArH), 7.23 (d, *J* = 8.4 Hz, 2H, ArH), 4.37–4.34 (m, 1H, CH), 2.96–2.93 (m, 2H, CH_2_), 2.37 (s, 3H, CH_3_); IR (KBr) ν: 3171, 3067, 1767, 1730, 1668, 1598 cm^−1^. Anal calcd. for C_19_H_18_N_4_O_3_S: C 59.67, H 4.74, N 14.65; Found: C 59.58, H 4.67, N 14.48.

*N*-((4-((2,4-dioxoimidazolidin-5-yl)methyl)phenyl)carbamothioyl)-4-methoxybenzamide **8e**, white solid, yield 69%, m.p. 224–226 ºC, ^1^H NMR (DMSO-d_6_, 300 MHz) δ: 12.74 (s, 1H, NH), 11.39 (s, 1H, NH), 10.50 (s, 1H, NH), 8.02 (d, *J* = 8.7 Hz, 2H, ArH,), 7.99 (s, 1H, NH),7.64 (d, *J* = 8.4 Hz, 2H, ArH), 7.22 (d, *J* = 8.4 Hz, 2H, ArH), 7.07 (d, *J* = 8.7 Hz, 2H, ArH), 4.37–4.34 (m, 1H, CH), 3.86 (s, 3H, OCH_3_), 2.95–2.93 (m, 2H, CH_2_); IR (KBr) ν: 3176, 3051, 1766, 1726, 1667, 1596 cm^−1^. Anal calcd. for C_19_H_18_N_4_O_4_S: C 57.27, H 4.55, N 14.16; Found: C 57.22, H 4.55, N 14.03.

*N*-((4-((2,4-dioxoimidazolidin-5-yl)methyl)phenyl)carbamothioyl)-4-nitrobenzamide **8f**, yellow solid, yield 82%, m.p. 226–228 ºC, ^1^H NMR (DMSO-d_6_, 300 MHz) δ: 12.75 (s, 1H, NH), 11.47(s, 1H, NH), 10.44 (s, 1H, NH), 8.38–7.93 (m, 5H, ArH + NH), 7.35–7.20 (m, 4H, ArH), 4.38–4.33 (m, 1H, CH), 2.97–2.92 (m, 2H, CH_2_); IR (KBr) ν: 3112, 3047, 1768, 1728, 1667, 1592 cm^−1^. Anal calcd. for C_18_H_15_N_5_O_5_S: C 52.30, H 3.66, N 16.94; Found: C 52.23, H 3.68, N 16.85.

*N*-((4-((2,4-dioxoimidazolidin-5-yl)methyl)phenyl)carbamothioyl)-2-methylbenzamide **8g**, white solid, yield 56%, m.p. 212–214 ºC, ^1^H NMR (DMSO-d_6_, 300 MHz) δ: 12.56 (s, 1H, NH), 11.68 (s, 1H, NH), 10.48 (s, 1H, NH), 7.96 (s, 1H, NH), 7.67 (d, *J* = 8.4 Hz, 2H, ArH), 7.51~7.22 (m, 6H, ArH), 4.37–4.34 (m, 1H, CH), 2.96–2.91 (m, 2H, CH_2_), 2.42 (s, 3H, CH_3_); IR (KBr) ν: 3181, 3066, 1767, 1722, 1673, 1595 cm^−1^. Anal calcd. for C_19_H_18_N_4_O_3_S: C 59.67, H 4.74, N 14.65; Found: C 59.69, H 4.75, N 14.74.

*N*-((4-((2,4-dioxoimidazolidin-5-yl)methyl)phenyl)carbamothioyl)-2-chlorobenzamide **8h**, white solid, yield 60%, m.p. 216–218 ºC, ^1^H NMR (DMSO-d_6_, 300 MHz) δ: 12.36 (s, 1H, NH), 11.98 (s, 1H, NH), 10.49 (s, 1H, NH), 7.97 (s, 1H, NH), 7.67–7.43 (m, 6H, ArH), 7.23 (d, *J* = 8.5 Hz, 2H, ArH), 4.37–4.34 (m, 1H, CH), 2.99–2.93 (m, 2H, CH_2_); IR (KBr) ν: 3194, 3060, 1766, 1717, 1679, 1592, 1537 cm^−1^. Anal calcd. for C_18_H_15_ClN_4_O_3_S: C 53.67, H 3.75, N 13.91; Found: C 53.60, H 3.75, N 13.86.

*N*-((4-((2,4-dioxoimidazolidin-5-yl)methyl)phenyl)carbamothioyl)-2-methoxybenzamide **8i**, white solid, yield 60%, m.p. 216–218 ºC, ^1^H NMR (DMSO-d_6_, 300 MHz) δ: 12.57 (s 1H, NH), 12.21 (s, 1H, NH), 10.49 (s, 1H, NH), 7.97 (s, 1H, NH), 7.94–7.91 (m, 1H, ArH), 7.70–7.64 (m, 3H, ArH), 7.31–7.14 (m, 4H, ArH), 4.37–4.34 (m, 1H, CH), 4.01 (s, 3H, OCH_3_), 2.96–2.94 (m, 2H, CH_2_); IR (KBr) ν: 3219, 3036, 1769, 1716, 1665, 1595 cm^−1^. Anal calcd. for C_19_H_18_N_4_O_4_S: C 57.27, H 4.55, N 14.16; Found: C 57.51, H 4.53, N 14.14.

*N*-((4-((2,4-dioxoimidazolidin-5-yl)methyl)phenyl)carbamothioyl)-2-nitrobenzamide **8j**, yellow solid, yield 50%, m.p. 222–224 ºC, ^1^H NMR (DMSO-d_6_, 300 MHz) δ: 12.27 (s, 1H, NH), 12.12 (s, 1H, NH), 10.49 (s, 1H, NH), 8.24–8.21 (m, 1H, ArH), 7.96 (s, 1H, NH), 7.94–7.77 (m, 3H, ArH), 7.66 (d, 2H, *J* = 8.4 Hz, ArH), 7.24 (d, 2H, *J* = 8.4 Hz, ArH), 4.38–4.34 (m, 1H, CH), 2.97–2.95 (m, 2H, CH_2_); IR (KBr) ν: 3219, 3036, 1769, 1716, 1665, 1595 cm^−1^. Anal calcd. for C_18_H_15_N_5_O_5_S: C 52.30, H 3.66, N 16.94; Found: C 52.25, H 3.76, N 16.84.

*N*-((4-((2,4-dioxoimidazolidin-5-yl)methyl)phenyl)carbamothioyl)-3-methylbenzamide **8k**, white solid, yield 64%, m.p. 220–221 ºC, ^1^H NMR (DMSO-d_6_, 300 MHz) δ: 12.64 (s, 1H, NH), 11.48 (s, 1H, NH), 10.49 (s, 1H, NH), 7.97 (s, 1H, NH), 7.83–7.63 (m, 4H, ArH), 7.49–7.40 (m, 2H, ArH), 7.23 (d, *J* = 8.4 Hz, 2H, ArH), 4.37–4.34 (m, 1H, CH), 2.97–2.92 (m, 2H, CH_2_), 2.36 (s, 3H, CH_3_); IR (KBr) ν: 3169, 3056, 1767, 1726, 1668, 1596 cm^−1^. Anal calcd. for C_19_H_18_N_4_O_3_S: C 59.67, H 4.74, N 14.65; Found: C 59.62, H 4.75, N 14.54.

*N*-((4-((2,4-dioxoimidazolidin-5-yl)methyl)phenyl)carbamothioyl)-3-nitrobenzamide **8l**, little yellow solid, yield 79%, m.p. 168–170 ºC, ^1^H NMR (DMSO-d_6_, 300 MHz) δ: 12.43 (s, 1H, NH), 12.01 (s, 1H, NH), 10.49 (s, 1H, NH), 8.78 (s, 1H, ArH), 8.51–8.36 (m, 2H, ArH), 7.99 (s, 1H, NH), 7.86–7.68 (m, 3H, ArH), 7.24 (d, *J* = 8.4 Hz, 2H, ArH), 4.38–4.34 (m, 1H, CH), 2.97–2.91 (m, 2H, CH_2_); IR (KBr) ν: 3180, 3056, 1770, 1719, 1669, 1602 cm^−1^. Anal calcd. for C_18_H_15_N_5_O_5_S: C 52.30, H 3.66, N 16.94; Found: C 52.32, H 3.60, N 16.84.

*N*-((4-((2,4-dioxoimidazolidin-5-yl)methyl)phenyl)carbamothioyl)thiophene-2-carboxamide **8m**, white solid, yield 82%, m.p. 244–246 ºC, ^1^H NMR (DMSO-d_6_, 300 MHz) δ: 12.46 (s, 1H, NH), 11.58 (s, 1H, NH), 10.48 (s, 1H, NH), 8.39–8.37 (m, 1H, ThH), 8.06–8.04 (m, 1H, ThH), 7.96 (s, 1H, NH), 7.63 (d, *J* = 8.5 Hz, 2H, ArH), 7.27–7.21 (m, 3H, ArH + ThH), 4.37–4.32 (m, 1H, CH), 2.96–2.91 (m, 2H, CH_2_); IR (KBr) ν: 3171, 3061, 1762, 1716, 1654, 1596 cm^−1^. Anal calcd. for C_16_H_14_N_4_O_3_S_2_: C 51.32, H 3.77, N 14.96; Found: C 51.60, H 3.81, N 14.96.

2-Chloro-*N*-((4-((2,4-dioxoimidazolidin-5-yl)methyl)phenyl)carbamothioyl)isonicotinamide **8n**, white solid, yield 80%, m.p. 202–204 ºC, ^1^H NMR (DMSO-d_6_, 300 MHz) δ: 12.25 (s, 1H, NH), 11.92 (s, 1H, NH), 10.48 (s, 1H, NH), 8.61 (d, *J* = 5.1 Hz, 1H, PyH), 7.99–7.82 (m, 3H, PyH + NH), 7.63 (d, *J* = 8.3 Hz, 2H, ArH), 7.24 (d, *J* = 8.3 Hz, 2H, ArH), 4.37–4.34 (m, 1H, CH), 2.99–2.89 (m, 2H, CH_2_); IR (KBr) ν: 3150, 3042, 1765, 1719, 1666, 1593 cm^−1^. Anal calcd. for C_17_H_14_ClN_5_O_3_S: C 50.56, H 3.49, N 17.34; Found: C 50.70, H 3.57, N 17.27.

2-(2,4-dichlorophenoxy)-*N*-((4-((2,4-dioxoimidazolidin-5-yl)methyl)phenyl)carbamothioyl)acetamide **8o**, white solid, yield 71%, m.p. 262–264 ºC, ^1^H NMR (DMSO-d_6_, 300 MHz) δ: 11.95 (s, 1H, NH), 10.41 (s, 1H, NH), 10.14 (s, 1H, NH), 7.91 (s, 1H, NH), 7.50 (d, *J* = 8.5 Hz, 2H, ArH), 7.40~7.08 (m, 4H, ArH), 4.83 (s, 2H, CH_2_), 4.36–4.30 (m, 1H, CH), 2.90–2.81 (m, 2H, CH_2_); IR (KBr) ν: 3159, 3048, 1762, 1732, 1669, 1597 cm^−1^. Anal calcd. for C_19_H_16_Cl_2_N_4_O_4_S: C 48.83, H 3.45, N 11.99; Found: C 48.72, H 3.55, N 11.92.

2-(4-dichlorophenoxy)-*N*-((4-((2,4-dioxoimidazolidin-5-yl)methyl)phenyl)carbamothioyl)acetamide **8p**, white solid, yield 70%, m.p. 210–212 ºC, ^1^H NMR (DMSO-d_6_, 300 MHz) δ: 11.94 (s, 1H, NH), 10.42 (s, 1H, NH), 10.06 (s, 1H, NH), 7.92 (s, 1H, NH), 7.52 (d, *J* = 8.5 Hz, 2H, ArH), 7.39~7.33 (m, 2H, ArH), 7.14~7.10 (m, 2H, ArH), 7.04~6.99 (m, 2H, ArH), 4.69 (s, 2H, CH_2_), 4.34–4.28 (m, 1H, CH), 2.93–2.85 (m, 2H, CH_2_); IR (KBr) ν: 3116, 3043, 1769, 1709, 1687, 1598 cm^−1^. Anal calcd. for C_19_H_17_ClN_4_O_4_S: C 52.72, H 3.96, N 12.94; Found: C 52.62, H 3.86, N 12.90.

*N*-((4-((4-oxo-2-thioxoimidazolidin-5-yl)methyl)phenyl)carbamothioyl)benzamide **9a** little yellow solid, yield 75%, m.p. 240–242 ºC, ^1^H NMR (DMSO-d_6_, 300 MHz) δ: 12.62 (s, 1H, NH), 11.51 (s, 2H, NH), 10.09 (s, 1H, NH), 7.99~7.96 (m, 2H, ArH), 7.69~7.52 (m, 5H, ArH), 7.24~7.21 (m, 2H, ArH), 4.60–4.56 (m, 1H, CH), 3.01–2.96 (m, 2H, CH_2_); IR (KBr) ν: 3185, 3081, 1772, 1742, 1655, 1597 cm^−1^. Anal calcd. for C_18_H_16_N_4_O_2_S_2_: C 56.23, H 4.19, N 14.57; Found: C 56.16, H 4.21, N 14.55.

*N*-((4-((4-oxo-2-thioxoimidazolidin-5-yl)methyl)phenyl)carbamothioyl)-4-flurobenzamide **9b** little yellow solid, yield 56%, m.p. 238–240 ºC, ^1^H NMR (DMSO-d_6_, 300 MHz) δ: 12.57 (s, 1H, NH), 11.59 (s, 1H, NH), 11.51 (s, 1H, NH), 10.10 (s, 1H, NH), 8.09~8.04 (m, 2H, ArH), 7.67~7.64 (m, 2H, ArH), 7.41~7.35 (m, 2H, ArH), 7.24~7.21 (m, 2H, ArH), 4.60–4.56 (m, 1H, CH), 3.01–2.95 (m, 2H, CH_2_); IR (KBr) ν: 3309, 3047, 1775, 1754, 1658, 1593 cm^−1^. Anal calcd. for C_18_H_15_FN_4_O_2_S_2_: C 53.72, H 3.76, N 13.92; Found: C 53.53, H 3.88, N 13.91.

*N*-((4-((4-oxo-2-thioxoimidazolidin-5-yl)methyl)phenyl)carbamothioyl)-4-chlorobenzamide **9c** little yellow solid, yield 58%, m.p. 240–242 ºC, ^1^H NMR (DMSO-d_6_, 300 MHz) δ: 12.52 (s, 1H, NH), 11.64 (s, 1H, NH), 11.50 (s, 1H, NH), 10.10 (s, 1H, NH), 8.00~7.97 (m, 2H, ArH), 7.67~7.60 (m, 4H, ArH), 7.24~7.21 (m, 2H, ArH), 4.60–4.56 (m, 1H, CH), 3.01–2.96 (m, 2H, CH_2_); IR (KBr) ν: 3158, 3077, 1776, 1732, 1617, 1597 cm^−1^. Anal calcd. for C_18_H_15_ClN_4_O_2_S_2_: C 51.61, H 3.61, N 13.37; Found: C 51.46, H 3.72, N 13.47.

*N*-((4-((4-oxo-2-thioxoimidazolidin-5-yl)methyl)phenyl)carbamothioyl)-4-methylbenzamide **9d** little yellow solid, yield 70%, m.p. 237–238 ºC, ^1^H NMR (DMSO-d_6_, 300 MHz) δ: 12.68 (s, 1H, NH), 11.51 (s, 1H, NH), 11.43 (s, 1H, NH), 10.10 (s, 1H, NH), 7.92~7.89 (m, 2H, ArH), 7.67~7.64 (m, 2H, ArH), 7.36~7.33 (m, 2H, ArH), 7.24~7.21 (m, 2H, ArH), 4.59–4.56 (m, 1H, CH), 3.01–2.95 (m, 2H, CH_2_), 2.40 (s, 3H, CH_3_); IR (KBr) ν: 3284, 3047, 1773, 1752, 1655, 1596 cm^−1^. Anal calcd. for C_19_H_18_N_4_O_2_S_2_: C 57.27, H 4.55, N 14.06; Found: C 57.20, H 4.65, N 14.11.

*N*-((4-((4-oxo-2-thioxoimidazolidin-5-yl)methyl)phenyl)carbamothioyl)-4-methoxybenzamide **9e** little yellow solid, yield 46%, m.p. 232–234 ºC, ^1^H NMR (DMSO-d_6_, 300 MHz) δ: 12.74 (s, 1H, NH), 11.50 (s, 1H, NH), 11.35 (s, 1H, NH), 10.10 (s, 1H, NH), 8.03~8.00 (m, 2H, ArH), 7.67~7.64 (m, 2H, ArH), 7.23~7.20 (m, 2H, ArH), 7.08~7.05 (m, 2H, ArH), 4.59–4.56 (m, 1H, CH), 3.86 (s, 3H, OCH_3_), 3.01–2.96 (m, 2H, CH_2_); IR (KBr) ν: 3306, 3051, 1774, 1752, 1668, 1596 cm^−1^. Anal calcd. for C_19_H_18_N_4_O_3_S_2_: C 55.05, H 4.38, N 13.52; Found: C 54.82, H 4.45, N 13.49.

*N*-((4-((4-oxo-2-thioxoimidazolidin-5-yl)methyl)phenyl)carbamothioyl)-4-nitrobenzamide **9f** little yellow solid, yield 85%, m.p. 244–245 ºC, ^1^H NMR (DMSO-d_6_, 300 MHz) δ: 12.40 (s, 1H, NH), 11.91 (s, 1H, NH), 11.50 (s, 1H, NH), 10.10 (s, 1H, NH), 8.36~8.33 (m, 2H, ArH), 8.18~8.15 (m, 2H, ArH), 7.68~7.64 (m, 2H, ArH), 7.25~7.21 (m, 2H, ArH), 4.60–4.56 (m, 1H, CH), 3.05–2.97 (m, 2H, CH_2_); IR (KBr) ν: 3202, 3060, 1775, 1746, 1672, 1594 cm^−1^. Anal calcd. for C_18_H_15_N_5_O_4_S_2_: C 50.34, H 3.52, N 16.31; Found: C 50.18, H 3.67, N 16.24.

*N*-((4-((4-oxo-2-thioxoimidazolidin-5-yl)methyl)phenyl)carbamothioyl)-2-methylbenzamide **9g** little yellow solid, yield 63%, m.p. 220–222 ºC, ^1^H NMR (DMSO-d_6_, 300 MHz) δ: 12.58 (s, 1H, NH), 11.68 (s, 1H, NH), 11.50 (s, 1H, NH), 10.10 (s, 1H, NH), 7.70~7.66 (m, 2H, ArH), 7.51~7.41 (m, 2H, ArH), 7.32~7.27 (m, 2H, ArH), 7.24~7.20 (m, 2H, ArH), 4.59–4.56 (m, 1H, CH), 3.02–2.95 (m, 2H, CH_2_), 2.42 (s, 3H, CH_3_); IR (KBr) ν: 3187, 3094, 1774, 1745, 1676, 1587 cm^−1^. Anal calcd. for C_19_H_18_N_4_O_2_S_2_: C 57.27, H 4.55, N 14.06; Found: C 57.14, H 4.62, N 14.35.

*N*-((4-((4-oxo-2-thioxoimidazolidin-5-yl)methyl)phenyl)carbamothioyl)-2-chlorobenzamide **9h** little yellow solid, yield 53%, m.p. 216–218 ºC, ^1^H NMR (DMSO-d_6_, 300 MHz) δ: 12.38 (s, 1H, NH), 11.98 (s, 1H, NH), 11.51 (s, 1H, NH), 10.10 (s, 1H, NH), 7.68~7.43 (m, 6H, ArH), 7.24~7.21 (m, 2H, ArH), 4.60–4.56 (m, 1H, CH), 3.02–2.96 (m, 2H, CH_2_); IR (KBr) ν: 3224, 3062, 1776, 1745, 1685, 1593 cm^−1^. Anal calcd. for C_18_H_15_ClN_4_O_2_S_2_: C 51.61, H 3.61, N 13.37; Found: C 51.44, H 3.70, N 13.36.

*N*-((4-((4-oxo-2-thioxoimidazolidin-5-yl)methyl)phenyl)carbamothioyl)-2-methoxybenzamide **9i** little yellow solid, yield 69%, m.p. 244–246 ºC, ^1^H NMR (DMSO-d_6_, 300 MHz) δ: 12.58 (s, 1H, NH), 11.50 (s, 1H, NH), 11.20 (s, 1H, NH), 10.09 (s, 1H, NH), 7.94~7.91 (m, 1H, ArH), 7.70~7.64 (m, 3H, ArH), 7.31~7.15 (m, 4H, ArH), 4.59–4.56 (m, 1H, CH), 4.01 (s, 3H, OCH_3_), 3.02–2.94 (m, 2H, CH_2_); IR (KBr) ν: 3159, 3084, 1775, 1752, 1651, 1595 cm^−1^. Anal calcd. for C_19_H_18_N_4_O_3_S_2_: C 55.05, H 4.38, N 13.52; Found: C 54.92, H 4.47, N 13.63.

*N*-((4-((4-oxo-2-thioxoimidazolidin-5-yl)methyl)phenyl)carbamothioyl)-2-nitrobenzamide **9j** little yellow solid, yield 54%, m.p. 202–204 ºC, ^1^H NMR (DMSO-d_6_, 300 MHz) δ: 12.29 (s, 1H, NH), 12.12 (s, 1H, NH), 11.51 (s, 1H, NH), 10.11 (s, 1H, NH), 8.24~8.21 (m, 1H, ArH), 7.94~7.88 (m, 1H, ArH), 7.81~7.66 (m, 4H, ArH), 7.25~7.22 (m, 2H, ArH), 4.61–4.56 (m, 1H, CH), 3.02–2.96 (m, 2H, CH_2_); IR (KBr) ν: 3166, 3030, 1775, 1740, 1693, 1598 cm^−1^. Anal calcd. for C_18_H_15_N_5_O_4_S_2_·1/3H_2_O: C 49.64, H 3.63, N 16.08; Found: C 49.67, H 3.68, N 16.19.

*N*-((4-((4-oxo-2-thioxoimidazolidin-5-yl)methyl)phenyl)carbamothioyl)-3-methylbenzamide **9k** little yellow solid, yield 88%, m.p. 234–236 ºC, ^1^H NMR (DMSO-d_6_, 300 MHz) δ: 12.65 (s, 1H, NH), 11.50 (s, 1H, NH), 11.47 (s, 1H, NH), 10.10 (s, 1H, NH), 7.83~7.65 (m, 4H, ArH), 7.49~7.40 (m, 2H, ArH), 7.24~7.21 (m, 2H, ArH), 4.60–4.56 (m, 1H, CH), 3.01–2.94 (m, 2H, CH_2_), 2.39 (s, 3H, CH_3_); IR (KBr) ν: 3176, 3051, 1776, 1751, 1657, 1597 cm^−1^. Anal calcd. for C_19_H_18_N_4_O_2_S_2_: C 56.42, H 4.65, N 13.85; Found: C 56.50, H 4.63, N 14.11.

*N*-((4-((4-oxo-2-thioxoimidazolidin-5-yl)methyl)phenyl)carbamothioyl)-3-nitrobenzamide **9l** little yellow solid, yield 73%, m.p. 242–243 ºC, ^1^H NMR (DMSO-d_6_, 300 MHz) δ: 12.43 (s, 1H, NH), 11.97 (s, 1H, NH), 11.51 (s, 1H, NH), 10.10 (s, 1H, NH), 8.78~8.76 (m, 1H, ArH), 8.50~8.47 (m, 1H, ArH), 8.38~8.35 (m, 1H, ArH), 7.86~7.81 (m, 1H, ArH), 7.68~7.65 (m, 2H, ArH), 7.25~7.22 (m, 2H, ArH), 4.60–4.56 (m, 1H, CH), 3.02–2.96 (m, 2H, CH_2_); IR (KBr) ν: 3172, 3090, 1774, 1738, 1692, 1604 cm^−1^. Anal calcd. for C_18_H_15_N_5_O_4_S_2_: C 50.34, H 3.52, N 16.31; Found: C 49.94, H 3.68, N 16.26.

*N*-((4-((4-oxo-2-thioxoimidazolidin-5-yl)methyl)phenyl)carbamothioyl)thiophene-2-carboxamide **9m** little yellow solid, yield 68%, m.p. 242–244 ºC, ^1^H NMR (DMSO-d_6_, 300 MHz) δ: 12.48 (s, 1H, NH), 11.58 (s, 1H, NH), 11.51 (s, 1H, NH), 10.10 (s, 1H, NH), 8.39–8.37 (m, 1H, ThH), 8.06–8.04 (m, 1H, ThH), 7.66~7.63 (m, 2H, ArH), 7.27~7.20 (m, 3H, ArH + ThH), 4.60–4.55 (m, 1H, CH), 3.01–2.95 (m, 2H, CH_2_); IR (KBr) ν: 3176, 3107, 1776, 1737, 1668, 1599 cm^−1^. Anal calcd. for C_16_H_14_N_4_O_2_S_3_: C 49.21, H 3.61, N 14.35; Found: C 49.16, H 3.72, N 14.52.

### 3.3. Bioassay of Herbicidal and Fungicidal Activity

The preliminary herbicidal activities of compounds **7**–**9** against *B. campestris* and *E. crus-galli* were assayed using the protocols in the references [11–13]. The preliminary fungicidal activities of compounds **7**–**9** against *F. oxysporurm*, *A. solani*, *B. berengeriana*, *C. arachidcola* and *F. graminearum* were evaluated using methods in the references [7,10] by the mycelium growth rate test [38]. The culture was incubated at 25 ± 0.5 ºC. Three replicates were performed and the mean measurements were calculated from the three replicates.

The greenhouse test was performed using the procedures in reference [39] according to the pre-emergence and post-emergence applications. The formulations were sprayed before the seedlings were planted in a pot or the formulations were sprayed during one to two-leaf appeared after the seedlings were planted in a pot. Then they were kept in the greenhouse to observe the root and stem growth of the plants in three weeks, and the inhibition rates of compounds were obtained comparison the fresh plant weights with the blank control. Three replicates were performed and the mean measurements were calculated from the three replicates.

## 4. Conclusions

The novel acylthiourea derivatives with hydantoin or thiohydantoin were synthesized in moderate to excellent yields using 5-(4-aminophenyl)- and 5-(4-aminobenzyl)-hydantoin or 5-(4-aminobenzyl)- thiohydantoin as raw materials and characterized by IR, ^1^H NMR spectra and elementary analysis. The preliminary bioassay showed that these compounds exhibit some herbicidal selectivity with the 91%, 94% and 87% inhibition rates of **7l**, **8o** and **8p** against *B. campestris*, and 100%, 100% and 95% efficacy against *B. campestris* in a greenhouse test, respectively. Compounds **7a**, **7b**, **7c** and **7d** exhibited 74%, 79%, 79% and 71% inhibition rates against *F. oxysporum*, respectively.

## Figures and Tables

**Table 1:** The herbicidal activities (inhibition rate, %) of compounds **7**, **8** and **9**.

Compd.	*B. campestris*	*E. crus-galli*	Compd.	*B. campestris*	*E. crus-galli*	Compd.	*B. campestris*	*E. crus-galli*
7a	20	10	8a	2	15	9a	3	10
7b	38	0	8b	12	15	9b	44	10
7c	0	15	8c	16	20	9c	40	5
7d	39	0	8d	14	5	9d	33	15
7e	29	10	8e	0	5	9e	0	15
7f	0	5	8f	25	0	9f	0	10
7g	49	10	8g	4	10	9g	6	25
7h	23	15	8h	10	10	9h	29	25
7i	0	0	8i	11	15	9i	18	25
7j	14	5	8j	25	0	9j	6	10
7k	6	20	8k	0	10	9k	0	5
7l	91	0	8l	0	0	9l	0	10
			8m	0	0	9m	33	5
			8n	7	10			
			8o	94	10			
			8p	87	10			

**Table 2:** The herbicidal activities (efficacy, %) of compounds **7l**, **8o** and **8p** in greenhouse test.

Compd.	*B. campestris*		*E. crus-galli*	
	Pre-emergence	Post-emergence	Pre-emergence	Post-emergence
**7l**	23	100	0	0
**8o**	46	100	0	10
**8p**	31	95	10	15

**Table 3:** The fungicidal activities (inhibition rate, %) of compounds **7** against several plant fungi.

Compd.	*F. oxysporum*	*A. Solani*	*B. berengeriana*	*C. arachidcola*	*F. graminearum*
**7a**	74	20	32	0	25
**7b**	79	24	23	0	54
**7c**	79	24	23	0	40
**7d**	71	21	7	6	44
**7e**	59	17	30	6	25
**7f**	24	27	28	6	10
**7g**	24	27	37	6	18
**7h**	71	3	20	0	40
**7i**	24	13	25	6	16
**7j**	24	27	37	6	18
**7k**	41	20	23	0	40
**7l**	15	17	40	0	12
Carbendazin	100	44	97	8	100
